# Sleep Disparities Across Pregnancy: A Michigan Cohort Study

**DOI:** 10.1089/whr.2023.0009

**Published:** 2023-05-15

**Authors:** Chia-Lun Yang, Erica C. Jansen, Galit Levi Dunietz, Kelly Hirko, Louise M. O'Brien, Jean M. Kerver

**Affiliations:** ^1^ Department of Epidemiology and Biostatistics, Michigan State University, East Lansing, Michigan, USA.; ^2^ Department of Nutritional Sciences, School of Public Health, University of Michigan, Ann Arbor, Michigan, USA.; ^3^ Division of Sleep Medicine, Department of Neurology, University of Michigan, Ann Arbor, Michigan, USA.; ^4^ Department of Obstetrics and Gynecology, and University of Michigan, Ann Arbor, Michigan, USA.; ^5^ Department of Oral and Maxillofacial Surgery, University of Michigan, Ann Arbor, Michigan, USA.

**Keywords:** disparities, pregnancy, trimester, sleep, socioeconomic

## Abstract

**Introduction:**

Poor sleep health during pregnancy is related to adverse pregnancy outcomes. This study aims to identify sociodemographic characteristics associated with sleep health during pregnancy and to examine how they relate to changes in sleep during pregnancy.

**Materials and Methods:**

Participants (*n* = 458) were from the Michigan Archive for Research on Child Health, which is a prospective pregnancy cohort. Sociodemographic characteristics and self-reported sleep timing and quality were collected in phone interviews. This longitudinal study collected sleep parameters once during the early trimesters and once during the third trimester. Fall asleep and wake-up times were used to calculate sleep duration and sleep midpoint.

**Results:**

Compared to the third trimester, sleep duration was 12 minutes longer (*p* = 0.02), fall asleep time was 21 minutes earlier (*p* < 0.001), and the midpoint of sleep was 12 minutes earlier (*p* = 0.01) in early trimesters. Shorter sleep duration was noted in younger women. Sleep midpoint was later in those who were younger, overweight, or obese, racial minorities, unmarried, and with lower educational levels or socioeconomic status, and who smoked before pregnancy after adjusting for covariates. After controlling for confounders, women who were not working for pay had higher likelihood of reduced sleep duration, and women who were unmarried were more likely to have a delayed sleep midpoint in the third trimester compared to the early trimesters.

**Conclusions:**

This study suggests that sleep parameters changed during pregnancy and sleep health differed by sociodemographic characteristics. Understanding sleep disparities could help with early detection of at-risk populations during prenatal care.

## Introduction

In the United States, it is widely documented that racial and socioeconomic disparities exist with regard to maternal health and pregnancy outcomes.^1,2^ Specifically, non-Hispanic Black women have a higher likelihood of experiencing hypertensive disease during pregnancy and preterm birth, and delivering a small for gestational age baby.^1^ These adverse pregnancy outcomes contribute to neonatal mortality and public health burden.^3,4^ Reducing disparities in health is a public health issue and is one of the goals of Healthy People 2020.^5^ Although there are myriad reasons for pregnancy disparities, emerging research suggests that poor sleep health might be a modifiable contributor.^6^

Short sleep duration, poor sleep quality, and late sleep timing (measured by midpoint) are commonly reported in pregnant women^7,8^ and may be risk factors for adverse perinatal outcomes, including gestational diabetes, cesarean sections, preterm delivery, and low birth weight.^9,10^ Specifically, both short sleep duration (<6 hours) and poor sleep quality (Pittsburgh Sleep Quality Index [PSQI] score >5) increase the risk of gestational diabetes.^10^ Women with poor sleep quality during late pregnancy experienced longer labor duration and were more likely to have cesarean sections.^11^ Nine to 10 hours of sleep in Japanese pregnant women was associated with lower incidence of low birth weight and small for gestational age infants.^12^ Sleep midpoint is the median of sleep onset and offset, and has been used to assess chronotype.^13^

Chronotype is a person's sleep and wake time preference, which is categorized into morning-type (those who prefer early sleep onset and/or offset and often with early sleep midpoint), evening-type (those who prefer late sleep onset and/or offset and typically with late sleep midpoint), and intermediate-type (not morning nor evening type) chronotype.^14^ US cohort studies suggest that later sleep midpoint (>5 AM) was related to a higher risk of gestational diabetes and a higher rate of preterm birth among nulliparous women.^15,16^ Evening-type chronotype (late sleep midpoint) was related to increased food cravings and the likelihood of gaining weight during early pregnancy.^17^ Furthermore, evening-type chronotype is related to lower consumption of vitamins and minerals,^18^ which are important for infant growth. Given the adverse outcomes of poor sleep health, sleep disparities among pregnant women warrant further study.

Literature has examined sleep disparities in pregnant women, with a focus on sleep duration and sleep problems. Evidence from the National Health and Nutrition Examination Survey and cohort studies suggests that pregnant women who identified as Black were more likely to experience short sleep (<6 hours) than people who identified as White,^19,20^ which could be due to the fact that poverty and obesity were more prevalent in this population.^21^ To our knowledge, only one prior study examined sleep midpoint in pregnant women. Findings from this multisite prospective cohort study of nulliparous women with a singleton birth during 16–21 weeks of gestation suggested that later sleep midpoint was related to younger age, race/ethnicity minorities, being underweight, holding a government/self-pay insurance, and prepregnancy smoking.^22^

Research on sleep disparities needs to acknowledge that sleep patterns change during pregnancy.^23^ One study showed that compared to prepregnancy, total amount of sleep increased during the first trimester, but sleep duration decreased during the second and third trimesters.^23^ Yet, it is unknown which groups may be at a higher risk of experiencing changes in sleep across gestation. Therefore, this study aimed to identify disparities in sleep health during pregnancy and in sleep changes over pregnancy in a large, racially diverse, state-wide cohort of pregnant women from Michigan. We hypothesized that overall sleep health decreases when pregnancy progresses, and that Black race/ethnicity and lower socioeconomic status (SES) would be associated with worse sleep health and with more adverse changes in sleep health across pregnancy.

## Materials and Methods

### Participants

Data are from the Michigan Archive for Research on Child Health (MARCH), a prospective, ongoing, statewide population-based pregnancy cohort. MARCH comprised a stratified random sample of births in Michigan's lower Peninsula. The overarching aim of the MARCH cohort is to understand impacts of maternal characteristics and behaviors during pregnancy on maternal and child health outcomes. Written informed consents were conducted in one of 21 prenatal care clinics across the state.

Birth certificate data were obtained, with explicit permission from participants, from the Michigan Department of Health and Human Services, Division for Vital Records and Health Statistics. Participants' sociodemographic characteristics and sleep patterns were obtained from the first two prenatal phone interviews. For this analysis, data were only considered if two time points (≤27 weeks; >27 weeks of gestation) of sleep parameters were collected. This analysis was approved by the Biomedical and Health Institutional Review Board (BIRB) at Michigan State University (LEGACY16-1429M; LEGACYC07-1201; STUDY00007014).

### Sleep parameters

Self-reported sleep parameters, including timing and quality, were collected once in the first or second trimester (early trimesters; ≤27 weeks) and once in the third trimester (>27 weeks). Participants reported their sleep timing by this question, “In the past week, what time did you usually fall asleep/wake up?,” which was used to calculate sleep duration and sleep midpoint. Sleep midpoint is calculated by the median time point between fall asleep and wake-up time and serves as an indicator of chronotype.^13^

Specifically, morning-type individuals tend to have an early sleep midpoint, and evening-type individuals tend to have a late sleep midpoint. Sleep midpoint later than 5 AM was considered late sleep midpoint.^15,16^ To examine whether participants changed their sleep during pregnancy, the differences in sleep duration and midpoint between two time points were calculated. Changes in sleep duration were categorized into “reduced sleep duration ≥30 mins” and “reduced sleep <30 mins.” Participants with reduced sleep duration ≥30 minutes had 30 minutes (or more) shorter sleep duration in the third trimester than in their early trimesters.

Changes in sleep midpoint were categorized into “delayed sleep midpoint ≥30 mins” and “delayed sleep midpoint <30 mins.” Individuals with delayed sleep midpoint for at least 30 minutes had 30 minutes (or more) later sleep midpoint in the third trimester than the early trimesters. These categories were made based on the distribution of variables. Participants reported their sleep quality by this question, “Comparing the quality of your sleep during this pregnancy to the year prior to pregnancy, would you say that the quality of your sleep is much better, somewhat better, about the same, somewhat worse, or much worse than before?.” The responses were collapsed into two categories: better or about the same, and worse than before pregnancy.

### Sociodemographic characteristics/covariates

Self-reported data on age (continuous), height (continuous), prepregnancy weight (continuous), race (white, Black, or others), educational level (less than high school; high school graduate, diploma, or general education development; some college/technical/associates; bachelor's degree; and graduate degree), marital status (married or living with a partner; divorced, separated, widowed, or never married), household income (<$50,000/year or ≥$50,000/year), job status (full time; part time; or not working for pay), health plan (from job, spouse, parents, or other; from the government), smoking before pregnancy (no or yes), alcohol consumption during pregnancy (no or yes), and parity (nulliparous, primiparous, or multiparous) were collected.

Health plan was included in this study as another indicator (surrogate) of household income since income variable had more missing data. Maternal age was categorized into quartiles: quartile 1 (<26); quartile 2 (26–29); quartile 3 (30–33); and quartile 4 (≥34). Body weight status was operationalized into two categories: normal weight or underweight (body mass index [BMI] ≤24.9) and overweight or obese (BMI ≥25).

### Data analysis

Analysis of variance and Student *t* test were used to assess mean differences in sleep parameters among groups with different sociodemographic characteristics, and linear regression models were used to assess mean differences in sleep parameters, while adjusted for covariates. Paired *t* tests were used to examine differences in sleep parameters, including sleep duration and timing between early trimesters and the third trimester. Prevalence ratios (an analog of risk ratios)^24^ of changes in sleep duration and sleep midpoint were analyzed by Poisson regression with robust error variance.^25^ Adjusted models were run to account for potential confounders, which were selected using causal frameworks^26^ and prior knowledge on the relationships between outcomes and sociodemographic characteristics described from the literature.^27–32^

Chi-square tests were used to assess changes in sleep quality across pregnancy, and to examine the characteristics of participants who were included in and excluded from the analysis to evaluate potential bias. A *p*-value <0.05 was considered statistically significant. Analyses were conducted using SAS 9.4 software (SAS Institute Inc., Cary, NC).

## Results

### Participants

**FIG. 1.**
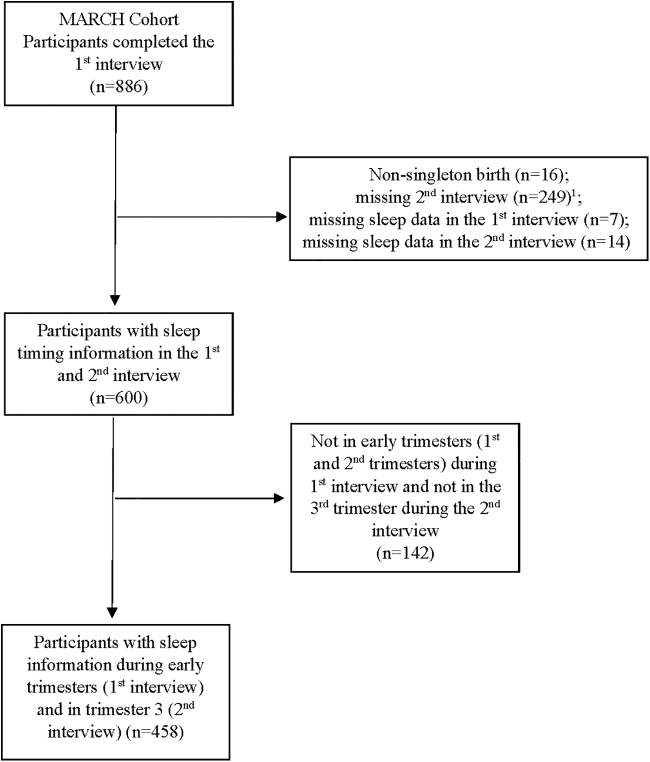
Flowchart for the inclusion of study participants. Second interview could be missing due to the fact that pregnancy was not yet in the later stage and therefore participants had not yet been contacted to complete the second interview. MARCH, Michigan Archive for Research on Child Health.^1^

A total of 886 participants enrolled and completed the first interview. As shown in Figure 1, participants were excluded if they had a nonsingleton birth (*n* = 16), were missing second interview (*n* = 249), were missing sleep measures at first (*n* = 7) or second interviews (*n* = 14), and were not in early trimesters during the first interview or not in the third trimester during the second interview (*n* = 142). A total of 458 pregnant women were included in this analysis. Participants' mean (standard deviation [SD]) gestational weeks was 14.5 (5.3) in the first interview and 32.9 (3.1) in the second interview.

Over half of the participants were overweight or obese in prepregnancy (58%); were white (65%); had more than high school education (75%); were married or living with a partner (76%); had household income of more than $50,000 per year (60%); had a full time job (60%); had a health plan from their job, spouse, parents, or others (58%); did not smoke before pregnancy (81%); did not drink during pregnancy (91%), and were primiparous or multiparous (61%) (Table 1). Sociodemographic characteristics differed between women included and excluded from this analysis (Supplementary Table S1). Study participants included in this analysis were more likely to identify as older, white adults, having a higher education level, married, higher income, working a full-time job, having a private health plan, and not having smoking behavior than those excluded from the analysis.

**Table 1.** Characteristics of Pregnant Women in the Michigan Archive for Research in Child Health Study (*n* = 458)

Variable	Value
Maternal age, mean (SD), years	29.8 (5.6)
Prepregnancy BMI, mean (SD), kg/m^2^	28.5 (8.1)
Prepregnancy weight status (BMI in kg/m^2^), *n* (%)	
Normal weight or underweight (<25)	191 (42.1)
Overweight or obese (≥25)	263 (57.9)
Race, *n* (%)	
White	295 (64.7)
Black	127 (27.9)
Other	34 (7.5)
Maternal educational level, *n* (%)	
Less than high school	43 (9.4)
High school graduate, diploma, or GED	73 (16.0)
Some college/technical/associates’	133 (29.2)
Bachelors' degree	87 (19.1)
Graduate degree	120 (26.3)
Marital/cohabitation status, *n* (%)	
Married or living with a partner	346 (75.7)
Divorced, separated, widowed, or never married	111 (24.3)
Household income, *n* (%), $	
<50,000	155 (39.8)
≥50,000	234 (60.2)
Job status, *n* (%)	
Full time	275 (60.2)
Part time	76 (16.6)
Not working for pay	106 (23.2)
Health plan, *n* (%)	
From job, spouse, parents, or other	262 (58.4)
From the government	187 (41.6)
Smoking before pregnancy, *n* (%)	
No	368 (80.5)
Yes	89 (19.5)
Alcohol consumption during pregnancy, *n* (%)	
No	415 (91.2)
Yes	40 (8.8)
Parity, *n* (%)	
Nulliparous	150 (39.3)
Primiparous or multiparous	232 (60.7)

Missing data: maternal age (*n* = 1); prepregnancy BMI (*n* = 4); race (*n* = 2); maternal educational level (*n* = 2); marital status (*n* = 1); household income (*n* = 69); job status (*n* = 1); health plan (*n* = 9); smoking (*n* = 1); alcohol consumption during pregnancy (*n* = 3); parity (*n* = 76).

BMI, body mass index; GED, general education development; SD, standard deviation.

### Sleep and sociodemographic characteristics

Sleep duration differed according to sociodemographic characteristics (Table 2). Women who were older and who were primiparous or multiparous had the shortest sleep duration during early trimesters. Participants who were older, had the highest educational level (graduate degree), and had private health plans experienced the shortest sleep duration in late gestation. However, associations between sleep duration and parity, education, and health plan were no longer significant after adjusting for covariates. Age was the only factor that associated with sleep duration; to illustrate, women who were at least 34 years old had over an hour shorter sleep duration than women who were less than 26 years old (*p* < 0.001, for both time points).

**Table 2.** Sleep Duration and Sleep Midpoint Across Pregnancy Among Different Groups

Variable^†^	Early trimesters (14.5 average gestation weeks)		Third trimester (32.9 average gestation weeks)	
	Mean (SD)^‡^	Adjusted differences in minutes (95% CI)§	Mean (SD)^‡^	Adjusted differences in minutes (95% CI)§
Sleep duration, hours				
Overall	8.5 (1.7)		8.3 (1.7)	
Maternal age, years				
Quartile 1 (<26)	9 (2.1)^a^	Ref.	8.8 (1.9)^a^	Ref.
Quartile 2 (26 to <30)	8.5 (1.6)^a,b^	−32.1 (−59.2 to −5)^*^	8.5 (1.7)^a,b^	−22.1 (−47.7 to 3.6)
Quartile 3 (30 to <34)	8.4 (1.5)^a,b^	−32.9 (−58.9 to −7)^*^	8.2 (1.4)^b,c^	−38.6 (−63.2 to −14.1)^*^
Quartile 4 (≥34)	8 (1.6)^b^	−62.6 (−89.3 to −35.8)^*^	7.6 (1.5)^d^	−73 (−98.3 to −47.6)^*^
Prepregnancy BMI category (kg/m^2^)				
Normal weight or underweight (≤24.9)	8.5 (1.6)	Ref.	8.4 (1.5)	Ref.
Overweight or obese (≥25)	8.4 (1.9)	−4.8 (−25.8 to 16.1)	8.2 (1.8)	−12 (−31.9 to 7.9)
Race				
White	8.5 (1.4)	Ref.	8.2 (1.4)	Ref.
Black	8.4 (2.5)	−7.1 (−28.9 to 14.8)	8.3 (2.2)	7.7 (−13.1 to 28.6)
Other	8.7 (1.4)	9.6 (−27.7 to 46.9)	8.7 (1.3)	29.5 (−6.1 to 65.1)
Maternal educational level				
Less than high school	8.9 (2.6)	27.1 (−12.6 to 66.7)	8.4 (2.7)^a,b^	5.7 (−31.7 to 43.1)
High school graduate, diploma, or GED	8.7 (2.2)	17.7 (−16.9 to 52.3)	8.5 (1.8)^a,b^	8.6 (−24 to 41.3)
Some college/technical/associates’	8.5 (1.9)	7 (−20.9 to 34.8)	8.5 (1.7)^a^	18.5 (−7.7 to 44.7)
Bachelors degree	8.2 (1)	−12.5 (−41.1 to 16)	8.1 (1.1)^a,b^	3.4 (−23.4 to 30.3)
Graduate degree	8.2 (1.3)	Ref.	7.9 (1.3)^b^	Ref.
Marital/cohabitation status				
Married or living with a partner	8.4 (1.4)	Ref.	8.2 (1.5)	Ref.
Divorced, separated, widowed, or never married	8.6 (2.6)	−1.1 (−29.7 to 27.5)	8.6 (2.1)	11 (−15.9 to 38)
Household income, $				
<50,000	8.5 (2.1)	−3.8 (−34 to 26.5)	8.3 (2.0)	−9.4 (−38.8 to 20)
≥50,000	8.3 (1.2)	Ref.	8.1 (1.3)	Ref.
Health plan				
From job, spouse, parents, or other	8.4 (1.3)	Ref.	8.1 (1.4)^a^	Ref.
From the government	8.6 (2.2)	−0.9 (−27.8 to 25.9)	8.5 (2.0)^b^	7.4 (−18.1 to 32.9)
Job status				
Full time	8.3 (1.7)	Ref.	8.3 (1.6)	Ref.
Part time	8.7 (2)	11.5 (−17.1 to 40)	8.2 (1.9)	−14.2 (−40.7 to 12.4)
Not working for pay	8.6 (1.8)	6.3 (−19.5 to 32.1)	8.4 (1.7)	−1 (−25.1 to 23)
Smoking before pregnancy				
No	8.4 (1.7)	Ref.	8.3 (1.6)	Ref.
Yes	8.6 (2)	−5.6 (−33.3 to 22.1)	8.2 (1.9)	−25.2 (−51.2 to 0.8)
Alcohol consumption during pregnancy				
No	8.5 (1.7)	Ref.	8.3 (1.6)	Ref.
Yes	8 (2.3)	−22.5 (−56.4 to 11.4)	7.9 (2.3)	−13.7 (−46.1 to 18.8)
Parity				
Nulliparous	8.7 (1.6)^a^	Ref.	8.5 (1.5)	Ref.
Primiparous or multiparous	8.2 (1.7)^b^	−20.1 (−42.5 to 2.4)	8.2 (1.6)	−5.6 (−26.5 to 15.3)
Midpoint of sleep, hh:mm				
Overall	2:51 (1:43)		3:03 (1:43)	
Maternal age, years				
Quartile 1 (<26)	3:21 (2:04)^a^	Ref.	3:25 (1:39)	Ref.
Quartile 2 (26 to <30)	2:36 (1:23)^b^	−45.2 (−71.9 to −18.4)^*^	3:03 (2:10)	−22 (−49.2 to 5.2)
Quartile 3 (30 to <34)	2:48 (1:42)^a,b^	−33.4 (−59.1 to −7.8)^*^	2:49 (1:11)	−35.4 (−61.4 to −9.4)^*^
Quartile 4 (≥34)	2:35 (1:29)^b^	−46.3 (−72.7 to −19.8)^*^	2:59 (1:48)	−25.6 (−52.4 to 1.3)
Prepregnancy BMI category (kg/m^2^)				
Normal weight or underweight (≤24.9)	2:42 (1:33)	Ref.	2:49 (1:30)^a^	Ref.
Overweight or obese (≥25)	2:58 (1:49)	10.6 (−10.9 to 32)	3:13 (1:50)^b^	18.8 (0.3 to 37.2)^*^
Race				
White	2:40 (1:31)^a^	Ref.	2:49 (1:21)^a^	Ref.
Black	3:14 (2:09)^b^	33.5 (12.3 to 54.8)^*^	3:31 (2:17)^b^	41.5 (20.2 to 62.7)^*^
Other	2:52 (1:14)^a,b^	12.2 (−24 to 48.4)	3:20 (1:54)^a,b^	31.2 (−5 to 67.5)
Maternal educational level				
Less than high school	3:18 (1:42)^a^	39.9 (0.7 to 79.1)^*^	3:41 (1:51)^a^	48.9 (9.9 to 87.9)^*^
High school graduate, diploma, or GED	3:09 (2:20)^a,b^	31.1 (−3.1 to 65.4)	3:08 (2:04)^a–c^	15.8 (−18.3 to 49.8)
Some college/technical/associates’	3:06 (1:46)^a,b^	33 (5.5 to 60.5)^*^	3:24 (1:53)^a,b^	38.7 (11.3 to 66.1)^*^
Bachelor's degree	2:32 (1:17)^a,b^	6.3 (−21.9 to 34.5)	2:44 (1:22)^c^	7 (−21.1 to 35)
Graduate degree	2:25 (1:20)^b^	Ref.	2:36 (1:18)^c^	Ref.
Marital/cohabitation status				
Married or living with a partner	2:44 (1:34)^a^	Ref.	2:50 (1:24)^a^	Ref.
Divorced, separated, widowed, or never married	3:10 (2:04)^b^	−5.5 (−33.8 to 22.8)	3:43 (2:21)^b^	37.2 (9.3 to 65.2)^*^
Household income, $				
<50,000	3:06 (1:47)^a^	32.6 (4 to 61.2)^*^	3:32 (2:05)^a^	38.3 (7.2 to 69.5)^*^
≥50,000	2:25 (1:17)^b^	Ref.	2:35 (1:15)^b^	Ref.
Health plan				
From job, spouse, parents, or other	2:31 (1:21)^a^	Ref.	2:47 (1:30)^a^	Ref.
From the government	3:15 (1:57)^b^	19.7 (−6.1 to 45.5)	3:26 (1:58)^b^	4.3 (−22.2 to 30.8)
Job status				
Full time	2:38 (1:43)^a^	Ref.	2:51 (1:39)^a^	Ref.
Part time	3:01 (1:49)^a,b^	15.3 (−13.3 to 44)	3:24 (1:46)^b^	29.1 (3.7 to 54.5)^*^
Not working for pay	3:15 (1:33)^b^	22.3 (−3.6 to 48.3)	3:20 (1:49)^b^	30 (6.9 to 53)^*^
Smoking before pregnancy				
No	2:46 (1:45)^a^	Ref.	2:54 (1:41)^a^	Ref.
Yes	3:11 (1:32)^b^	10.7 (−16.6 to 38)	3:40 (1:45)^b^	37.2 (10.4 to 63.9)^*^
Alcohol consumption during pregnancy				
No	2:49 (1:41)	Ref.	3:01 (1:43)	Ref.
Yes	3:08 (2:00)	18.9 (−14.9 to 52.8)	3:31 (1:51)	23.6 (−9.8 to 57)
Parity				
Nulliparous	2:50 (1:41)	Ref.	2:59 (1:38)	Ref.
Primiparous or multiparous	2:52 (1:41)	−6.7 (−29.3 to 15.9)	2:59 (1:29)	−7.6 (−27.6 to 12.4)

† Missing data: maternal age (*n* = 1); prepregnancy BMI (*n* = 4); race (*n* = 2); maternal educational level (*n* = 2); marital status (*n* = 1); household income (*n* = 69); job status (*n* = 1); health plan (*n* = 9); smoking (*n* = 1); alcohol consumption during pregnancy (*n* = 3); parity (*n* = 76).

‡ Different letters denote significant differences between each other (*p* < 0.05) based on Student *t* tests (two categories) and analysis of variance (three or more categories).

§Adjusted differences were calculated from linear regression models that adjusted for different variables. Negative sleep midpoints represent earlier sleep midpoint and vice versa. Adjusted variables were as follows:

Age and race were not adjusted for covariates.

BMI: adjusted for race, education, marital status, job status, smoking, alcohol consumption, parity.

Education: adjusted for age and race.

Marital status: adjusted for age, race, and education.

Job status: adjusted for age, race, education, marital status, and parity.

Income, health plan, smoking, alcohol consumption, and parity: adjusted for age, race, education, marital status, and job status.

^*^ *p* < 0.05 in the linear regression models. CI does not encompass 0.

CI, confidence interval.

Sleep midpoint was related to several sociodemographic characteristics (Table 2). Participants with the latest midpoint of sleep in early trimesters were younger, were more likely to identify as Black, had a lower education level (less than high school), were more likely unmarried, had lower household income, had a government health plan, were not working for pay, and had smoking behavior before pregnancy. The associations between midpoint of sleep and certain sociodemographic characteristics (marital, health plan, job, and smoking status) were attenuated after adjusting for covariates in the linear regression models.

Sleep midpoint in the third trimester was latest in participants who were 30–33 years old, were overweight or obese, identified as Black, had a lower education level, were unmarried, had lower household income, had a health plan from the government, held a part-time job or were not working for pay, and were smokers. Health plan sources became an insignificant factor after controlling for covariates.

### Sleep patterns across pregnancy

Sleep parameters changed during pregnancy. About 13% of women slept less than 7 hours in early trimesters and the percentage increased to 16% in the third trimester (Supplementary Table S2). Participants with late sleep midpoint (later than 5 AM) increased from 7% in early trimesters to 8% in the third trimester (Supplementary Table S3). In early trimesters and the third trimester, mean (SD) sleep duration was 8.5 (1.7) hours and 8.3 (1.7) hours, fall asleep time was 22:34 (1:58) and 22:55 (1:52); sleep midpoint was 2:51 (1:43) and 3:03 (1:43); and wake-up time was 7:08 (2:06) and 7:12 (1:58), respectively.

**FIG. 2.**
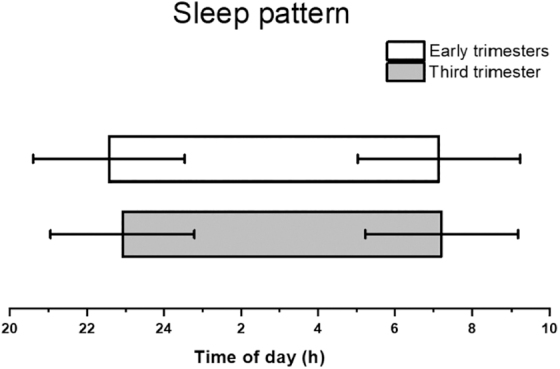
Sleep pattern between early trimesters and the third trimester. Sleep duration was longer (*p* = 0.02), fall asleep time was earlier (*p* < 0.001), and the midpoint of sleep was earlier (*p* = 0.01) in early trimesters compared to the third trimester. No significant difference in wake-up time was noted (*p* = 0.48). Participants' mean (standard deviation) gestational weeks were 14.5 (5.3) in the early trimesters and 32.9 (3.1) in the third trimester interviews.

Sleep duration was on average 12 minutes longer, fall asleep time was 21 minutes earlier, and the midpoint of sleep was 12 minutes earlier in early trimesters compared to the third trimester (Fig. 2). No significant difference in wake-up time was observed across pregnancy time points. Sleep quality became worse as pregnancy progressed, as 44% of individuals experienced worse sleep quality (compared to the year before pregnancy) in early trimesters, and this increased to 69% in the third trimester (*p* < 0.001).

### Prevalence ratio of reduced sleep duration

A total of 47% of women reduced sleep duration by at least 30 minutes. The only factor associated with changes in sleep duration was work status. A little over half (56%) of participants who were not working for pay reduced their sleep duration during pregnancy compared to 43% who were working (Table 3). This association remained after adjustment for confounders, such that women who were not working for pay were 37% more likely to have reduced sleep duration.

**Table 3.** Prevalence Ratio of Reduced Sleep Duration and Delayed Sleep Midpoint Among Different Sociodemographic Characteristics

Variable^*^	Reduced sleep duration (early trimester sleep ≥30 minutes longer than third trimester)			Delayed sleep midpoint (sleep midpoint in the third trimester is ≥30 minutes later than the early trimesters)		
	***n***/***N*** (%)^†^	Unadjusted prevalence ratio (95% CI)	Adjusted prevalence ratio (95% CI)^‡^	***n***/***N*** (%)§	Unadjusted prevalence ratio (95% CI)	Adjusted prevalence ratio (95% CI)^‡^
Maternal age, years						
Quartile 1 (<26)	58/117 (49.6)	Ref.		51/117 (43.6)	Ref.	
Quartile 2 (26 to <30)	42/105 (40)	0.8 (0.6–1.1)		42/105 (40)	0.9 (0.7–1.3)	
Quartile 3 (30 to <34)	59/125 (47.2)	1.0 (0.7–1.2)		48/125 (38.4)	0.9 (0.7–1.2)	
Quartile 4 (≥34)	57/110 (51.8)	1.0 (0.8–1.4)		38/110 (34.5)	0.8 (0.6–1.1)	
*p*		0.37			0.57	
Prepregnancy weight status (BMI in kg/m^2^)						
Normal weight or underweight (<25)	85/191 (44.5)	Ref.	Ref.	64/191 (33.5)	Ref.	Ref.
Overweight or obese (≥25)	128/263 (48.7)	1.1 (0.9–1.3)	1.2 (0.9–1.5)	112/263 (42.6)	1.3 (1.0–1.6)	1.3 (1–1.7)
*p*		0.38	0.13		0.05	0.08
Race						
White	149/295 (50.5)	Ref.		101/295 (34.2)	Ref.	
Black	53/127 (41.7)	0.8 (0.5–1.2)		62/127 (48.8)	1.4 (1.1–1.8)	
Other	13/34 (38.2)	0.8 (0.7–1)		15/34 (44.1)	1.3 (0.9–1.9)	
*p*		0.16			0.01	
Maternal educational level						
Less than high school	34/73 (46.6)	1.1 (0.8–1.6)	1.3 (0.9–1.9)	32/73 (43.8)	1.5 (1–2.3)	1.3 (0.8–2)
High school graduate, diploma, or GED	38/87 (43.7)	1 (0.7–1.3)	1.1 (0.8–1.6)	26/87 (29.9)	1.3 (0.9–1.9)	1.1 (0.7–1.7)
Some college/technical/associates	23/43 (53.5)	1 (0.8–1.3)	1.1 (0.8–1.4)	22/43 (51.2)	1.3 (0.9–1.8)	1.2 (0.8–1.6)
Bachelors degree	62/133 (46.6)	0.9 (0.7–1.2)	0.9 (0.7–1.2)	57/133 (42.9)	0.9 (0.6–1.4)	0.9 (0.6–1.3)
Graduate degree	57/120 (47.5)	Ref.	Ref.	40/120 (33.3)	Ref.	Ref.
*p*		0.88	0.57		0.07	0.53
Marital/cohabitation status						
Married or living with a partner	164/346 (47.4)	Ref.	Ref.	118/346 (34.1)	Ref.	Ref.
Divorced, separated, widowed, or never married	51/111 (45.9)	1 (0.8–1.2)	1 (0.8–1.4)	60/111 (54.1)	1.6 (1.3–2)	1.4 (1–1.9)
*p*		0.79	0.94		<0.001	0.03
Household income, $						
<50,000	75/155 (48.4)	1.0 (0.8–1.3)	1.1 (0.8–1.5)	78/155 (50.3)	1.6 (1.2–2)	1.2 (0.8–1.8)
≥50,000	109/234 (46.6)	Ref.	Ref.	74/234 (31.6)	Ref.	Ref.
*p*		0.73	0.70		<0.001	0.27
Job status						
Full time	117/275 (42.5)	Ref.	Ref.	101/275 (36.7)	Ref.	Ref.
Part time	39/76 (51.3)	1.2 (0.9–1.6)	1.2 (0.9–1.7)	36/76 (47.4)	1.3 (1–1.7)	1.1 (0.8–1.5)
Not working for pay	59/106 (55.7)	1.3 (1.1–1.6)	1.4 (1.1–1.8)	41/106 (38.7)	1.1 (0.8–1.4)	1.1 (0.8–1.5)
*p*		0.04	0.0496		0.21	0.77
Health plan						
From job, spouse, parents, or other	124/262 (47.3)	Ref.	Ref.	90/262 (34.4)	Ref.	Ref.
From the government	87/187 (46.5)	1 (0.8–1.2)	0.9 (0.7–1.2)	86/187 (46)	1.3 (1.1–1.7)	1 (0.8–1.4)
*p*		0.87	0.57		0.01	0.81
Smoking before pregnancy						
No	166/368 (45.1)	Ref.	Ref.	138/368 (37.5)	Ref.	Ref.
Yes	49/89 (55.1)	1.2 (1–1.5)	1.2 (0.9–1.5)	40/89 (44.9)	1.2 (0.9–1.6)	1.1 (0.8–1.4)
*p*		0.07	0.23		0.18	0.72
Alcohol consumption during pregnancy						
No	196/415 (47.2)	Ref.	Ref.	159/415 (38.3)	Ref.	Ref.
Yes	18/40 (45)	1 (0.7–1.4)	0.9 (0.7–1.4)	19/40 (47.5)	1.2 (0.9–1.8)	1.1 (0.8–1.6)
*p*		0.79	0.78		0.23	0.45
Parity						
Nulliparous	73/150 (48.7)	Ref.	Ref.	60/150 (40)	Ref.	Ref.
Primiparous or multiparous	100/232 (43.1)	0.9 (0.7–1.1)	0.8 (0.6–1)	89/232 (38.4)	1 (0.7–1.2)	1 (0.7–1.3)
*p*		0.28	0.06		0.75	0.72

^*^ Missing data: maternal age (*n* = 1); prepregnancy BMI (*n* = 4); race (*n* = 2); maternal educational level (*n* = 2); marital status (*n* = 1); household income (*n* = 69); job status (*n* = 1); health plan (*n* = 9); smoking (*n* = 1); alcohol consumption during pregnancy (*n* = 3); parity (*n* = 76).

† Number and percentage of participants who experienced reduced sleep duration.

‡ Adjusted variables were as follows:

Age and race were not adjusted for covariates.

BMI: adjusted for race, education, marital status, job status, smoking, alcohol consumption, parity.

Education: adjusted for age and race.

marital status: adjusted for age, race, and education.

Job status: adjusted for age, race, education, marital status, and parity.

Income, health plan, smoking, alcohol consumption, and parity: adjusted for age, race, education, marital status, and job status.

§Number and percentage of participants who experienced delayed sleep midpoint.

### Prevalence ratio of delayed sleep midpoint

Thirty-nine percent of women delayed sleep midpoint (≥30-minute differences). Prevalence ratios of delayed sleep midpoint during pregnancy were higher in women who identified as Black; were unmarried; had lower household income; and had a health plan from the government (Table 3). After adjusting for covariates, marital status remained a significant factor, such that women who were divorced, separated, widowed, or never married had 1.4 times the prevalence of delayed sleep midpoint compared to married women.

## Discussion

We report relationships between sleep duration, midpoint, and sociodemographic characteristics among pregnant women in a Michigan cohort study. After controlling for covariates, age was the only factor related to self-reported sleep duration. In contrast, later sleep midpoint was reported in racial minorities, women who were younger, were overweight or obese in prepregnancy, had lower overall SES, and who smoked before pregnancy. During pregnancy, sleep duration was reduced, sleep midpoint was delayed, and sleep quality decreased from early trimesters to the third trimesters. Currently not working for pay was related to reduced sleep duration during pregnancy, and unmarried status was related to delayed sleep midpoint. The evidence from this study supports that sociodemographic information, which is usually collected early in pregnancy, may be used to identify women at risk of poor sleep health, particularly late sleep timing, during pregnancy.

In this study, age, maternal education, health plans, and parity were related to prenatal sleep duration. However, the observed associations were no longer significant after adjusting for covariates, and older age appears to be the factor that was most strongly related to shorter sleep duration. The lack of associations is in contrast with previous studies.^20,22^

First, one US study with that objectively measured sleep duration of 782 nulliparous women with a singleton gestation observed that shorter sleep duration was related to racial/ethnic minority status and commercial insurance after adjustment for covariates.^22^ Beyond the differences in the measurement of sleep between the two studies, this study had a slightly older participant mean age (30 vs. 27 years), had more participants who identified as Black (28% vs. 11.8%), and included women who were primiparous or multiparous. Second, another study in Metro Detroit reported that Black race/ethnicity was related to shorter sleep duration among women with a gestational age of 28 weeks.^20^ The discrepancy in the findings could be due to the fact that this study included a larger sample size of diverse populations from a larger geographic area.

In this study, mean sleep duration significantly decreased by 12 minutes over pregnancy, which is consistent with findings from previous studies.^7,22^ For example, sleep duration decreased steadily during pregnancy (8.2 hours in the first trimester, 8.0 hours in the second trimester, and 7.8 hours in the third trimester) among Finnish women.^23^ Pregnancy-related symptoms, including frequent urination, discomfort, and pain, often increase in the third trimester,^7,33^ which could account for the decreases in sleep duration over pregnancy observed in our study. These data are not available in this analysis, so no direct comparison could be made. Also, diagnosed sleep disorders, including obstructive sleep apnea^34^ and restless leg syndrome,^7^ tend to increase during pregnancy. Thus, both pregnancy-related symptoms and diagnosed sleep disorders could contribute to decreased sleep duration during pregnancy.

Our study represents an extension to the literature since we also examined possible disparities in sleep duration changes over pregnancy. We observed that unemployment was related to reduced sleep duration (≥30 minutes). Unemployment has been associated with malnutrition, depression, and insomnia,^35^ and each of these factors may have contributed to further reducing sleep duration during the third trimester, when sleep difficulties become more prevalent.

Overall, our study findings suggest more pronounced disparities in sleep midpoint than sleep duration during pregnancy. Later sleep midpoint was observed in participants who were younger, identified as Black, had lower educational level, and had a lower household income in early trimesters and the third trimester in this study. Furthermore, women who were overweight or obese before pregnancy, had lower SES, and engaged in unhealthy lifestyle behaviors experienced later sleep midpoint in the third trimester.

Our findings in sleep timing were supported by one previous study, which examined disparities in sleep midpoint among pregnant women, and noted that later sleep midpoint at 16–21 gestational weeks was related to underweight or obese status, younger age, race/ethnicity minority status, governmental health insurance status, and engagement in smoking behaviors.^22^ There are multiple plausible mechanisms to explain disparities in sleep timing during pregnancy. First, age is a predictor of sleep midpoint because younger women generally had a later sleep timing than older women, with a peak in sleep midpoint around age 18, which gradually moves earlier with age.^36^ Second, people with evening-type chronotype (late sleep midpoint) tend to have a poorer diet quality^37^ and are more likely to consume unhealthy snacks, which increases risk of being overweight or obese^38^; both poor diet and overweight/obesity could further exacerbate sleep problems related to late bedtimes, including insomnia.^39,40^

Furthermore, people who are racial minorities are more likely to live in poverty, have obesity, and live in neighborhoods exposed to noise,^21^ all of which contribute to sleep problems and could therefore lead to later sleep timing.^20,41^ Lifestyle behaviors such as smoking have been related to evening-type chronotype and overall poor sleep in a prior study.^42^ The physiological implication of having a late sleep midpoint is that it can lead to late-night eating, which has been shown to shorten gestational length by 0.45 weeks and was associated with 2.2 times higher odds of preterm delivery.^43^ Late sleep midpoint has also being reported to increase the likelihood of gestational diabetes and preterm delivery.^8,16^ Therefore, identifying disparities in pregnant women could possibly help with reducing risk of adverse pregnancy outcomes.

In this study, participants' sleep midpoint was 12 minutes later, and fall asleep time was 21 minutes later in the third trimester, compared to early trimesters, but wake-up time did not change during pregnancy. This result was partially supported by one recent study that measured sleep midpoint before and across pregnancy,^44^ finding that median bedtime in pregnant women in the first (22:15) and second trimester (22:10) was earlier than before pregnancy (22:30), but bedtime in the third trimester was not different from before pregnancy.^44^

Our study adds to the literature by further examining who was at risk of delayed sleep midpoint. Results from this analysis demonstrated that married women were less likely to have a delayed sleep midpoint, with over half of unmarried participants delaying sleep midpoint by more than 30 minutes. These findings may be understood through a social framework perspective, which suggests a link between marital status and health, as spouses may be influencing their partner's health behaviors by monitoring and regulating habits.^45^ Also, the relationships may create a greater sense of personal responsibility for health.^45^

In addition to changes in sleep duration and timing, sleep quality changed during pregnancy in this study. Consistent with a prior study that observed sleep quality decreased from the second trimester to the third trimester,^44^ this study reported poorer sleep quality in the third trimester. Possible explanations for the decrease in quality, including pregnancy-related symptoms and sleep disorders, increased in late pregnancy.^7,46^ Due to changes in sleep across pregnancy, future studies on maternal sleep should consider collecting sleep information in several time points across pregnancy.

There were several strengths of this study. First, this is a longitudinal cohort study, which examined sleep in the same participants across pregnancy. Second, multiple sociodemographic characteristics were examined in this study. In addition, this analysis has a reasonable sample size. Some weaknesses should be noted. First, time awake during the night was not collected, so was not available for the analysis. Also, subjective sleep measurements are not as reliable as objective measurement, but the prior were normally comparable to the latter among healthy adults in recording sleep and wake cycles.^47^ Furthermore, collecting subjective sleep is efficient in population-based or community-based studies.^48^

We observed differences in age, race, education level, marital status, sociodemographic characteristics, and health behaviors among participants who were included and excluded from the analysis. This may limit the generalizability of our results to Michigan communities. In addition, we did not have information on napping, which can be frequent in pregnant women. Occupational information on whether participants were shift workers or not was not available in the survey. On a related note, we did not have access to a comprehensive survey on sleep quality. Nonetheless, a prior study used a one-item sleep quality scale to assess sleep quality and it was highly correlated with the PSQI score.^49^ Maternal psychological stress was not assessed in this study, and could potentially mediate the relationships between SES and sleep disparities.

## Conclusions

In this study, sleep duration and quality decreased and sleep timing was delayed over the course of pregnancy. Sleep duration did not differ according to sociodemographic characteristics, except for age, with younger women reporting longer sleep duration. Sleep midpoint was later in those who were younger, overweight or obese, racial minorities, unmarried, and with lower educational levels or SES, and who smoked during pregnancy. Overall, identifying pregnant people with poor sleep health could help with early detection of at-risk populations during prenatal care.

## Supplementary Material

Supplemental data

Supplemental data

Supplemental data

## Abbreviations Used

BMI: body mass index
CI: confidence interval
GED: general education development
MARCH: Michigan Archive for Research on Child Health
PSQI: Pittsburgh Sleep Quality Index
SD: standard deviation
SES: socioeconomic status
