# Supplementary material for: Sleep Disparities Across Pregnancy: A Michigan Cohort Study
Source: Womens Health Rep (New Rochelle). 2023 May 15;4(1):219–31. doi: 10.1089/whr.2023.0009 (PMC10210214; doi:10.1089/whr.2023.0009)
Supplement: Supplemental data [file Supp_TableS1.docx]

**Supplementary Material**

**Supplemental Table S1. Characteristics of participants included in and excluded from the analysis**

| **Variable** | **Included (n=458)**  **n/N (%)^1^** | **Excluded (n=428)**  **n/N (%)^2^** | **p** |
| --- | --- | --- | --- |
| **Maternal age, years** |  |  | <0.001 |
| Quartile 1 (<26) | 117 (44.7) | 145 (55.3) |  |
| Quartile 2 (26 to <30) | 105 (52.2) | 96 (47.8) |  |
| Quartile 3 (30 to <34) | 125 (61.6) | 78 (38.4) |  |
| Quartile 4 (≥34) | 110 (62.2) | 67 (37.9) |  |
| **Pre-pregnancy weight status, (BMI in kg/m^2^)** |  |  | 0.58 |
| Normal weight or underweight (<25) | 191 (53.1) | 169 (46.9) |  |
| Overweight or obese (≥25.0) | 263 (51.2) | 251 (48.8) |  |
| **Race** |  |  | <0.001 |
| White | 295 (57.2) | 221 (42.8) |  |
| Black | 127 (42.6) | 171 (57.4) |  |
| Other | 34 (54.8) | 28 (45.2) |  |
| **Maternal educational level** |  |  | <0.001 |
| Less than high school | 43 (47.8) | 47 (52.2) |  |
| High school graduate, diploma, or GED | 73 (38.8) | 115 (61.2) |  |
| Some college/technical/associates’ | 133 (43.5) | 173 (56.5) |  |
| Bachelors degree | 87 (63.5) | 50 (36.5) |  |
| Graduate Degree | 120 (77.9) | 34 (22.1) |  |
| **Marital/cohabitation status** |  |  | <0.001 |
| Married or living with a partner | 346 (56.5) | 266 (43.5) |  |
| Divorced, separated, widowed, or never married | 111 (42.1) | 153 (58) |  |
| **Household income, $** |  |  | <0.001 |
| <50,000 | 155 (42.5) | 210 (57.5) |  |
| ≥50,000 | 234 (64.8) | 127 (35.2) |  |
| **Job status** |  |  | <0.001 |
| Full time | 275 (56.8) | 209 (43.2) |  |
| Part time | 76 (45.5) | 91 (54.5) |  |
| Not working for pay | 106 (46.9) | 120 (53.1) |  |
| **Health Plan** |  |  | <0.001 |
| From job, spouse, parents, or other | 262 (62.7) | 156 (37.3) |  |
| From the government | 187 (42.2) | 256 (57.8) |  |
| **Smoking before pregnancy** |  |  | <0.001 |
| No | 368 (55.8) | 292 (44.2) |  |
| Yes | 89 (41.6) | 125 (58.4) |  |
| **Alcohol consumption during pregnancy** |  |  | 0.30 |
| No | 415 (52.9) | 369 (47.1) |  |
| Yes | 40 (47.1) | 45 (52.9) |  |
| **Parity** |  |  | 0.50 |
| Nulliparous | 150 (66.1) | 77 (33.9) |  |
| Primiparous or multiparous | 232 (63.2) | 135 (36.8) |  |

BMI: body mass index.

^1^Missing data: Maternal age (n=1); Pre-pregnancy BMI (n=4); Race (n=2); Maternal educational level (n=2); Marital status (n=1); Household income (n=69); Job status (n=1); Health plan (n=9); Smoking (n=1); Alcohol consumption during pregnancy(n=3); Parity (n=76).

^2^Missing data: Maternal age (n=42); Pre-pregnancy BMI (n=8); Race (n=8); Maternal educational level (n=9); Marital status (n=9); Household income (n=91); Job status (n=8); Health plan (n=16); Smoking (n=11); Alcohol consumption during pregnancy(n=14); Parity (n=216).

P values were from Chi-square tests.
