# Supplementary material for: Sleep Disparities Across Pregnancy: A Michigan Cohort Study
Source: Womens Health Rep (New Rochelle). 2023 May 15;4(1):219–31. doi: 10.1089/whr.2023.0009 (PMC10210214; doi:10.1089/whr.2023.0009)
Supplement: Supplemental data [file Supp_TableS2.docx]

**Supplementary Table S2: Sleep duration among different group of participants, n (%)**

|  | **Early trimesters** | | | |  | **Third trimester** | |  |  |  |
| --- | --- | --- | --- | --- | --- | --- | --- | --- | --- | --- |
| **Sleep duration** | **<7 h** | **7-9 h** | **9-11 h** | **>11 h** | **p** | **<7 h** | **7-9 h** | **9-11 h** | **>11 h** | **p** |
| **Overall** | 61 (13.3) | 205 (44.8) | 149 (32.5) | 43  (9.4) |  | 71 (15.5) | 216 (47.2) | 141 (30.8) | 30  (6.5) |  |
| **Maternal age, years** |  |  |  |  | <0.001 |  |  |  |  | <0.001 |
| Quartile 1 (<26) | 19 (16.2) | 32 (27.4) | 41  (35) | 25 (21.4) |  | 16 (13.7) | 38 (32.5) | 48  (41) | 15 (12.8) |  |
| Quartile 2 (26 to <30) | 11 (10.5) | 50 (47.6) | 37 (35.2) | 7  (6.7) |  | 13 (12.4) | 53 (50.5) | 30 (28.6) | 9  (8.6) |  |
| Quartile 3 (30 to <34) | 12  (9.6) | 65  (52) | 40  (32) | 8  (6.4) |  | 16 (12.8) | 60  (48) | 46 (36.8) | 3  (2.4) |  |
| Quartile 4 (≥34) | 18 (16.4) | 58 (52.7) | 31 (28.2) | 3  (2.7) |  | 26 (23.6) | 65 (59.1) | 16 (14.6) | 3  (2.7) |  |
| **Pre-pregnancy weight status, (BMI in kg/m^2^), n (%)** |  |  |  |  | 0.18 |  |  |  |  | 0.10 |
| Normal weight or underweight (<25) | 19  (10) | 96 (50.3) | 59 (30.9) | 17  (8.9) |  | 23 (12.04) | 91 (47.64) | 68 (35.6) | 9  (4.71) |  |
| Overweight or obese (≥25) | 41 (15.6) | 109 (41.4) | 87 (33.1) | 26  (9.9) |  | 46 (17.49) | 123 (46.77) | 73 (27.76) | 21 (7.98) |  |
| **Race** |  |  |  |  | <0.001 |  |  |  |  | <0.001 |
| White | 23  (7.8) | 152 (51.5) | 99 (33.6) | 21  (7.1) |  | 37 (12.5) | 158 (53.6) | 88 (29.8) | 12  (4.1) |  |
| Black | 35 (27.6) | 34 (26.8) | 39 (30.7) | 19 (15) |  | 31 (24.4) | 41 (32.3) | 38 (29.9) | 17 (13.4) |  |
| Other | 2  (5.9) | 18 (52.9) | 11 (32.4) | 3  (8.8) |  | 1  (2.9) | 17  (50) | 15 (44.1) | 1  (2.9) |  |
| **Maternal educational level** |  |  |  |  | <0.001 |  |  |  |  | <0.001 |
| Less than high school | 10 (23.3) | 11 (25.6) | 10 (23.3) | 12 (27.9) |  | 13 (30.2) | 11 (25.6) | 11 (25.6) | 8  (18.6) |  |
| High school graduate, diploma, or GED | 15 (20.6) | 20 (27.4) | 26 (35.6) | 12 (16.4) |  | 15 (20.6) | 22 (30.1) | 28 (38.4) | 8  (11) |  |
| Some college/technical/associates’ | 19 (14.3) | 47 (35.3) | 52 (39.1) | 15 (11.3) |  | 19 (14.3) | 53 (39.9) | 49 (36.8) | 12  (9) |  |
| Bachelors degree | 8  (9.2) | 55 (63.2) | 23 (26.4) | 1  (1.2) |  | 7 (8.1) | 56 (64.4) | 24 (27.6) | 0  (0) |  |
| Graduate Degree | 9  (7.5) | 70 (58.3) | 38 (31.7) | 3  (2.5) |  | 16 (13.3) | 73 (60.8) | 29 (24.2) | 2  (1.7) |  |
| **Marital/cohabitation status** |  |  |  |  | <0.001 |  |  |  |  | <0.001 |
| Married or living with a partner | 30  (8.7) | 181 (52.3) | 116 (33.5) | 19  (5.5) |  | 45  (13) | 185 (53.5) | 103 (29.8) | 13  (3.8) |  |
| Divorced, separated, widowed, or never married | 31 (27.9) | 23 (20.7) | 33 (29.7) | 24 (21.6) |  | 25 (22.5) | 31 (27.9) | 38 (34.2) | 17 (15.3) |  |
| **Household income, $** |  |  |  |  | <0.001 |  |  |  |  | <0.001 |
| <50,000 | 31  (20) | 52 (33.6) | 51 (32.9) | 21 (13.6) |  | 36 (23.2) | 53 (34.2) | 47 (30.3) | 19 (12.3) |  |
| ≥50,000 | 17  (7.3) | 134 (57.3) | 74 (31.6) | 9  (3.9) |  | 23  (9.8) | 144 (61.5) | 63 (26.9) | 4  (1.7) |  |
| **Health Plan** |  |  |  |  | <0.001 |  |  |  |  | <0.001 |
| From job, spouse, parents, or other | 24  (9.2) | 144 (55) | 81 (30.9) | 13  (5) |  | 32 (12.2) | 151 (57.6) | 71 (27.1) | 8  (3.1) |  |
| From the government | 34 (18.2) | 58  (31) | 67 (35.8) | 28  (15) |  | 35 (18.7) | 63 (33.7) | 68 (36.4) | 21 (11.2) |  |
| **Job status** |  |  |  |  | 0.54 |  |  |  |  | 0.88 |
| Full time | 38 (13.8) | 129 (46.9) | 88  (32) | 20  (7.3) |  | 46 (16.7) | 129 (46.9) | 84 (30.6) | 16  (5.8) |  |
| Part time | 11 (14.5) | 29 (38.2) | 26 (34.2) | 10 (13.2) |  | 12 (15.8) | 36 (47.4) | 23 (30.3) | 5  (6.6) |  |
| Not working for pay | 12 (11.3) | 46 (43.4) | 35  (33) | 13 (12.3) |  | 12 (11.3) | 51 (48.1) | 34 (32.1) | 9  (8.5) |  |
| **Smoking before pregnancy** |  |  |  |  | 0.02 |  |  |  |  | 0.01 |
| No | 45 (12.2) | 176 (47.8) | 117 (31.8) | 30  (8.2) |  | 49 (13.3) | 186 (50.5) | 111 (30.2) | 22  (6) |  |
| Yes | 16  (18) | 28 (31.5) | 32  (36) | 13 (14.6) |  | 21 (23.6) | 30 (33.7) | 30 (33.7) | 8  (9) |  |
| **Alcohol consumption during pregnancy** |  |  |  |  | 0.01 |  |  |  |  | 0.03 |
| No | 49 (11.8) | 194 (46.8) | 133 (32.1) | 39  (9.4) |  | 59 (14.2) | 200 (48.2) | 131 (31.6) | 25  (6) |  |
| Yes | 11 (27.5) | 10  (25) | 16  (40) | 3  (7.5) |  | 11 (27.5) | 15 (37.5) | 9  (22.5) | 5  (12.5) |  |
| **Parity** |  |  |  |  | 0.06 |  |  |  |  | 0.42 |
| Nulliparous | 15  (10) | 61 (40.7) | 58 (38.7) | 16 (10.7) |  | 17 (11.3) | 72  (48) | 50 (33.3) | 11  (7.3) |  |
| Primiparous or multiparous | 35 (15.1) | 113 (48.7) | 70 (30.2) | 14  (6) |  | 39 (16.8) | 110 (47.4) | 71 (30.6) | 12  (5.2) |  |

GED: general education development; BMI: body mass index.
