# Supplementary material for: Sleep Disparities Across Pregnancy: A Michigan Cohort Study
Source: Womens Health Rep (New Rochelle). 2023 May 15;4(1):219–31. doi: 10.1089/whr.2023.0009 (PMC10210214; doi:10.1089/whr.2023.0009)
Supplement: Supplemental data [file Supp_TableS3.docx]

**Supplementary Table S3: Sleep midpoint among different group of participants, n (%)**

|  | **Early trimesters** | |  | **Third trimester** | |  |
| --- | --- | --- | --- | --- | --- | --- |
| **Sleep midpoint** | **≤5 AM** | **>5 AM** | **p** | **≤5 AM** | **>5 AM** | **p** |
| **Overall** | 427 (93.2) | 31 (6.8) |  | 420 (91.7) | 38 (8.3) |  |
| **Maternal age, years** |  |  | <0.001 |  |  | 0.03 |
| Quartile 1 (<26) | 100 (85.5) | 17 (14.5) |  | 101 (86.3) | 16 (13.7) |  |
| Quartile 2 (26 to <30) | 101 (96.2) | 4 (3.8) |  | 95 (90.5) | 10 (9.5) |  |
| Quartile 3 (30 to <34) | 116 (92.8) | 9 (7.2) |  | 121 (96.8) | 4 (3.2) |  |
| Quartile 4 (≥34) | 109 (99.1) | 1 (0.9) |  | 102 (92.7) | 8 (7.3) |  |
| **Pre-pregnancy weight status, (BMI in kg/m^2^), n (%)** |  |  | 0.13 |  |  | 0.37 |
| Normal weight or underweight (<25) | 182 (95.3) | 9 (4.7) |  | 178 (93.2) | 13 (6.8) |  |
| Overweight or obese (≥25) | 241 (91.6) | 22 (8.4) |  | 239 (90.9) | 24 (9.1) |  |
| **Race** |  |  | 0.002 |  |  | 0.02 |
| White | 283 (95.9) | 12 (4.1) |  | 278 (94.2) | 17 (5.8) |  |
| Black | 110 (86.6) | 17 (13.4) |  | 109 (85.8) | 18 (14.2) |  |
| Other | 32 (94.1) | 2 (5.9) |  | 31 (91.2) | 3 (8.8) |  |
| **Maternal educational level** |  |  | 0.004 |  |  | 0.007 |
| Less than high school | 36 (83.7) | 7 (16.3) |  | 36 (83.7) | 7 (16.3) |  |
| High school graduate, diploma, or GED | 68 (93.2) | 5 (6.9) |  | 67 (91.8) | 6 (8.2) |  |
| Some college/technical/associates’ | 119 (89.5) | 14 (10.5) |  | 116 (87.2) | 17 (12.8) |  |
| Bachelors degree | 85 (97.7) | 2 (2.3) |  | 84 (96.6) | 3 (3.5) |  |
| Graduate Degree | 117 (97.5) | 3 (2.5) |  | 116 (96.7) | 4 (3.3) |  |
| **Marital/cohabitation status** |  |  | 0.001 |  |  | <0.001 |
| Married or living with a partner | 330 (95.4) | 16 (4.6) |  | 329 (95.1) | 17 (4.9) |  |
| Divorced, separated, widowed, or never married | 96 (86.5) | 15 (13.5) |  | 90 (81.1) | 21 (18.9) |  |
| **Household income, $** |  |  | <0.001 |  |  | <0.001 |
| <50,000 | 138 (89) | 17 (11) |  | 134 (86.5) | 21 (13.6) |  |
| ≥50,000 | 229 (97.9) | 5 (2.1) |  | 229 (97.9) | 5 (2.1) |  |
| **Health Plan** |  |  | <0.001 |  |  | <0.001 |
| From job, spouse, parents, or other | 255 (97.3) | 7 (2.7) |  | 250 (95.4) | 12 (4.6) |  |
| From the government | 164 (87.7) | 23 (12.3) |  | 161 (86.1) | 26 (13.9) |  |
| **Job status** |  |  | 0.09 |  |  | 0.74 |
| Full time | 262 (95.3) | 13 (4.7) |  | 253 (92) | 22 (8) |  |
| Part time | 69 (90.8) | 7 (9.2) |  | 68 (89.5) | 8 (10.5) |  |
| Not working for pay | 95 (89.6) | 11 (10.4) |  | 98 (92.5) | 8 (7.6) |  |
| **Smoking before pregnancy** |  |  | 0.65 |  |  | 0.02 |
| No | 344 (93.5) | 24 (6.5) |  | 343 (93.2) | 25 (6.8) |  |
| Yes | 82 (92.1) | 7 (7.9) |  | 76 (85.4) | 13 (14.6) |  |
| **Alcohol consumption during pregnancy** |  |  | 0.63 |  |  | 0.69 |
| No | 386 (93) | 29 (7) |  | 381 (91.8) | 34 (8.2) |  |
| Yes | 38 (95) | 2 (5) |  | 36 (90) | 4 (10) |  |
| **Parity** |  |  | 0.36 |  |  | 0.33 |
| Nulliparous | 138 (92) | 12 (8) |  | 137 (91.3) | 13 (8.7) |  |
| Primiparous or multiparous | 219 (94.4) | 13 (5.6) |  | 218 (94) | 14 (6) |  |

GED: general education development; BMI: body mass index.
